# Effects of the order of exposure to antimicrobials on the incidence of multidrug-resistant *Pseudomonas aeruginosa*

**DOI:** 10.1038/s41598-023-35256-8

**Published:** 2023-05-31

**Authors:** Nami Yasuda, Tomoko Fujita, Takahiro Fujioka, Mei Tagawa, Naoki Kohira, Kensho Torimaru, Sumiko Shiota, Takanori Kumagai, Daichi Morita, Wakano Ogawa, Tomofusa Tsuchiya, Teruo Kuroda

**Affiliations:** 1 Department of Microbiology, Graduate School of Medicine, Dentistry and Pharmaceutical Sciences, Okayama University, 1-1-1, Tsushima-Naka, Kita-ku, Okayama, 700-8530 Japan; 2 Department of Microbiology, Graduate School of Biomedical and Health Sciences, Hiroshima University, 1-2-3, Kasumi, Minami-ku, Hiroshima, 734-8553 Japan; 3 Department of Molecular Biology, School of Pharmacy, Shujitsu University, 1-6-1 Nishigawara, Naka-ku, Okayama, 703-8516 Japan; 4 Department of Microbiology and Biochemistry, Daiichi University of Pharmacy, 22-1, Tamagawa-Machi, Minami-ku, Fukuoka, 815-8511 Japan

**Keywords:** Antimicrobial resistance, Bacterial genes

## Abstract

Multidrug-resistant *Pseudomonas aeruginosa* (MDRP) is one of the most important pathogens in clinical practice. To clarify the mechanisms contributing to its emergence, we isolated MDRPs using the *P. aeruginosa* PAO1, the whole genome sequence of which has already been elucidated. Mutant strains resistant to carbapenems, aminoglycosides, and new quinolones, which are used to treat *P. aeruginosa* infections, were isolated; however, none met the criteria for MDRPs. Then, PAO1 strains were exposed to these antimicrobial agents in various orders and the appearance rate of MDRP varied depending on the order of exposure; MDRPs more frequently appeared when gentamicin was applied before ciprofloxacin, but were rarely isolated when ciprofloxacin was applied first. Exposure to ciprofloxacin followed by gentamicin increased the expression of MexCD-OprJ, an RND-type multidrug efflux pump, due to the NfxB mutation. In contrast, exposure to gentamicin followed by ciprofloxacin resulted in more mutations in DNA gyrase. These results suggest that the type of quinolone resistance mechanism is related to the frequency of MDRP and that the risk of MDRP incidence is highly dependent on the order of exposure to gentamicin and ciprofloxacin.

## Introduction

*Pseudomonas aeruginosa* is an opportunistic pathogen that exhibits high intrinsic resistance to antimicrobial agents. *P. aeruginosa* may acquire resistance through the inappropriate use and/or long-term administration of antimicrobial agents. Many multidrug-resistant *P. aeruginosa* strains (MDRPs) have been identified and are highly resistant to three antimicrobial agents, namely, broad-spectrum β-lactams, aminoglycosides, and fluoroquinolones^1–3^. Since the number of antibiotics that are effective against MDRP is limited, countermeasures are important.

To promote the appropriate use of antimicrobial agents, it is important to clarify the relationship between the antimicrobial agents used and the mechanisms underlying the acquisition of resistance. Analyses of clinical isolates have progressed in recent years, resulting in a more detailed understanding of MDRPs^1–3^. However, it is impossible to find the parental strain of an isolated MDRP in clinical settings because once the MDRP is isolated, the susceptible parent strain has already disappeared. Therefore, analyses of clinically isolated MDRPs alone cannot reveal a direct relationship between the type of antimicrobial used in treatment and the mechanism underlying the acquisition of resistance to it.

In the present study, we isolated and analyzed MDRPs using the *P. aeruginosa* PAO1 strain, the whole genomic DNA sequence of which is available. We were unable to isolate MDRPs with an exposure to one or two antimicrobial agents, but were successful with a sequential exposure to three antimicrobial agents. Therefore, MDRPs emerged by stacking multiple resistance mechanisms. We also found that the order in which antimicrobials are used may affect the emergence of MDRPs.

## Results

### Isolation of drug-resistant mutants from *P. aeruginosa* PAO1

We isolated drug-resistant mutants from *P. aeruginosa* PAO1 for carbenicillin (β-lactams), imipenem (carbapenems), gentamicin and amikacin (aminoglycosides), and ciprofloxacin and levofloxacin (fluoroquinolones), which are used to treat *P. aeruginosa* infection in clinical settings. The frequency of appearance of resistant mutants was 7.5×10^−7^ to 1.1×10^−8^ (Table 1).

**Table 1:** Number of *P. aeruginosa* mutants isolated from PAO1.

Antimicrobial agent	MIC for PAO1	Number of mutants isolated at each concentration				Total
	(μg/ml)	1 × MIC	2 × MIC	4 × MIC	8 × MIC	
Carbenicillin	32	N.D.	59	3	0	62
Imipenem	1	N.D.	29	40	0	69
Gentamicin	4	N.D.	N.D.	112	N.T.	112
Amikacin	4–8	N.D.	N.D.	23	N.T.	23
Ciprofloxacin	0.25	N.D.	N.D.	50	0	50
Levofloxacin	0.5–1	164	39	2	0	205
Erythromycin	256	N.D.	N.D.	19	0	19

N.D.; not determined because too many colonies appeared, N.T.; not tested.

**Figure 1:**
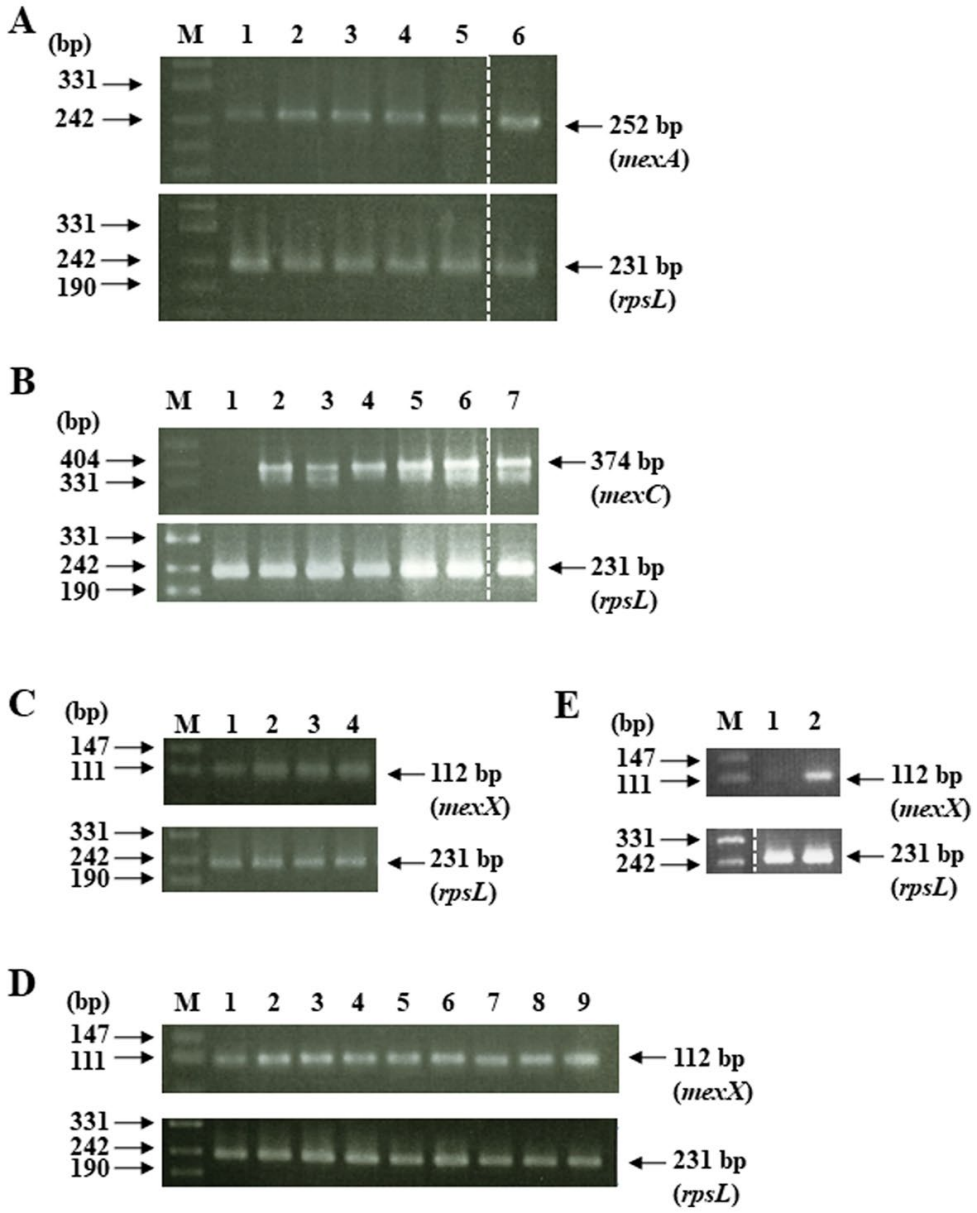
Expression of RND-type multidrug efflux pump genes. (**A**) Upper panel: *mexA*, Lower panel: *rpsL* (internal control), M: marker (pUC19/*Msp*I), 1: PAO1, 2: CAR204, 3: CAR401, 4: LV201, 5: LV206, 6: LV438. (**B**) Upper panel: *mexC*, Lower panel: *rpsL* (internal control), M: marker (pUC19/*Msp*I), 1: PAO1, 2: CIP101, 3: CIP126, 4: IC4430, 5: IC4404, 6: LV235, 7: LV801. (**C**) Upper panel: *mexX*, Lower panel: *rpsL* (internal control), M: marker (pUC19/*Msp*I), 1: PAO1, 2: AMK1606, 3: AMK1612, 4: GM458. (**D**) Upper panel: *mexX*, Lower panel: *rpsL* (internal control), M: marker (pUC19/*Msp*I), 1: PAO1, 2: GM458, 3: IG4455, 4: CG4401, 5: CG4411, 6: CgG4479, 7: ICG444391, 8: ICG444242, 9: CIG44408. (**E**) Upper panel: *mexX*, Lower panel: *rpsL* (internal control), M: marker (pUC19/*Msp*I), 1: PAO1, 2: CgIG44441. The white dashed line indicated a cut section of a photograph of electrophoresis. Raw data were shown in Supplemental data.

Among 540 mutants, the spectrum of antibiotic resistance was investigated in 92 randomly selected mutants. These mutants were categorized into seven groups by the spectrum (Table 2). Sixty-eight mutants exhibited multidrug resistance (groups 1–3). In these groups, the drug-resistant spectrum was the same or similar to the substrate pattern of RND-type efflux pumps^4–7^. In some mutants from each group, the expression of RND-type efflux pump genes was investigated by RT-PCR. The expression of *mexA* was up-regulated in group 1 mutants (Fig. 1A). The expression of *mexC* was observed in group 2 mutants, but was not detected in PAO1 (Fig. 1B). The expression of *mexX* was up-regulated in groups 3 and 4 (Fig. 1C). Since the mutant strains classified as group 5 were resistant only to carbenicillin, it is speculated that AmpC β-lactamase is highly expressed in these strains^8^. In group 6, only imipenem resistance was observed, and the down-regulated expression of the outer membrane porin OprD was speculated^9,10^. Group 7 mutants showed resistance to ciprofloxacin and levofloxacin, indicating a mutation in DNA gyrase or topoisomerase IV^11^. Other than imipenem mutants, the majority of mutants exhibited multidrug resistance, suggested the up-regulated expression of multidrug efflux pumps. However, no mutant considered to be MDRP (MIC for imipenem: 16 μg/ml or higher, amikacin: 32 μg/ml or higher, ciprofloxacin: 4 μg/ml or higher) was isolated using this procedure.

**Table 2:** Spectrum of antibiotic resistance for isolated mutants.

Group	Antimicrobial agent for which MIC increased	Putative resistance mechanism	Number of mutants	Antimicrobial agents used for mutant isolation
1	CAR, CIP, LVX, TET, CHL, ACR	Efflux via MexAB-OprM	9	CAR
			15	LVX
2	CIP, LVX, TET, CHL, ERY, ACR	Efflux via MexCD-OprJ	10	CIP
			7	LVX
			9	ERY
3	GEN, AMK, CIP	Efflux via MexXY-OprM	10	GEN
			8	AMK
4	GEN, AMK	Efflux via MexXY-OprM or mutations in ribosomes	3	AMK
5	CAR	Overexpression of AmpC	4	CAR
6	IPM	Depletion of OprD	15	IPM
7	CIP, LVX	Mutation in DNA gyrase	1	CIP
			1	LVX

ACR: acriflavine, AMK: amikacin, CAR: carbenicillin, CHL: chloramphenicol, CIP: ciprofloxacin, ERY: erythromycin, GEN: gentamicin,

IPM: imipenem, LVX: levofloxacin, TET: tetracycline.

Since the frequency of isolation of one antibiotic ranged between 10^–7^ and 10^–8^, difficulties were associated with the direct isolation of MDRPs from PAO1 following exposure to one drug. Therefore, we assumed that MDRPs may be isolated by sequential exposure to three drugs.

### Isolation of resistant mutants by a sequential exposure to 2 and 3 drugs

**Figure 2:**
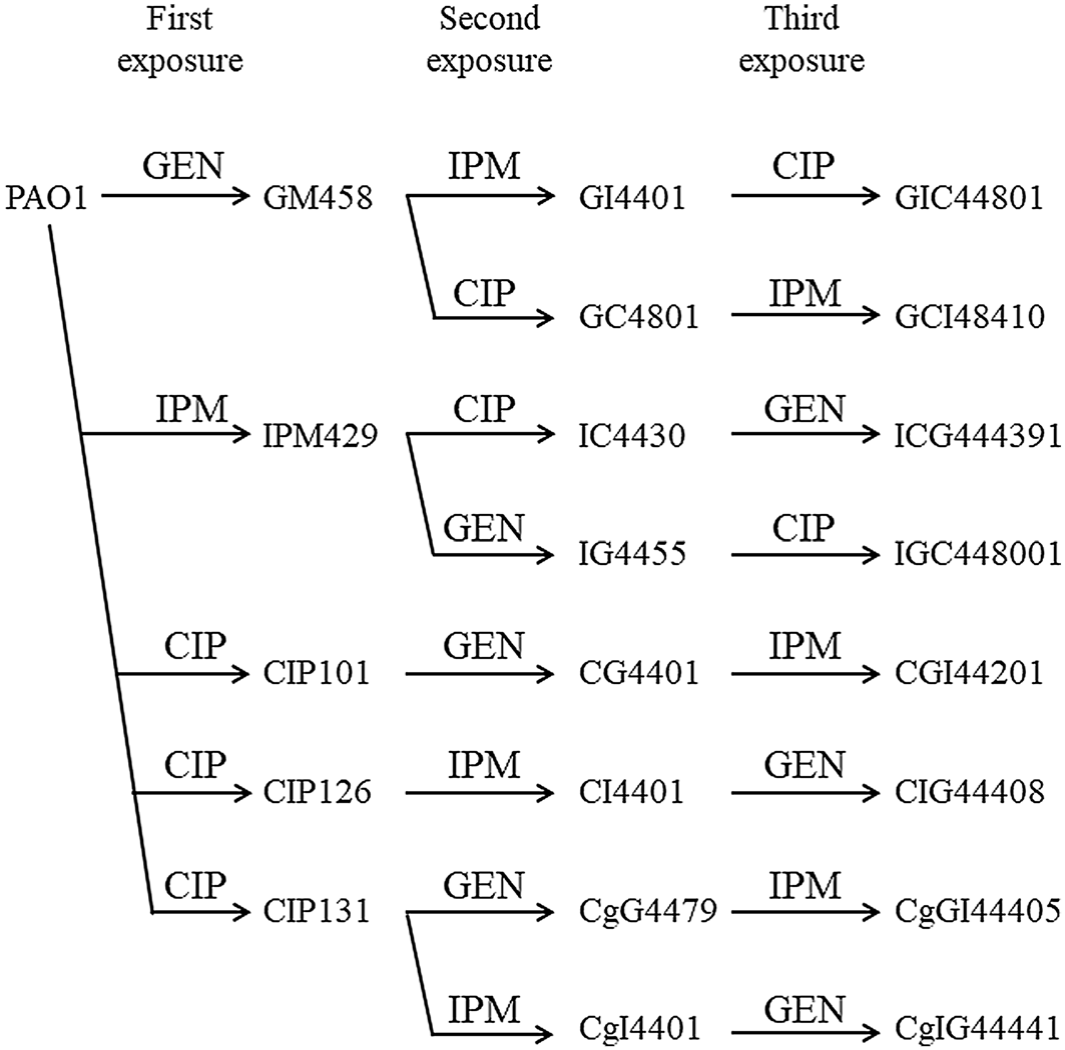
Flow of acquiring mutants. Using PAO1 as the parental strain, resistant mutants of gentamicin, imipenem, and ciprofloxacin were initially obtained. A representative strain was selected from among them and exposed to the second antimicrobial agent to obtain a resistant mutant strain. In the same way, the third antimicrobial agent was exposed to obtain three drug resistant mutants.

**Figure 3:**
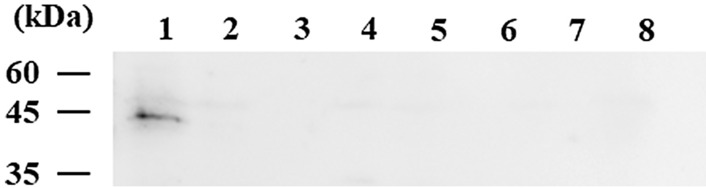
Western blotting analysis of OprD. 1: PAO1, 2: IPM429, 3: GI4401, 4: CI4401, 5: CgI4401, 6: GCI48410, 7: CGI44201, 8: CgGI44405. After chemiluminescence treatment, blots were visualized with a gel imaging system. Only the required lanes were shown here. Raw data before trimming were shown in Supplemental data.

Since MDRPs were not isolated by an exposure to only one drug, we attempted to isolate MDRPs by a sequential exposure to three different drugs (Fig. 2). IPM429, GM458, CIP101, CIP126, and CIP131 were used as mutants after the first exposure. The IPM429 mutant showed increased MIC for imipenem, and the porin protein OprD was not detected (Table 3, Fig. 3). Since some gentamicin-resistant mutants show an unstable resistance phenotype, further analyses were performed using GM458 mutants exhibiting stable resistance to gentamicin. GM458 showed increased MIC for gentamicin, amikacin, ciprofloxacin, and levofloxacin (Table 3) as well as the up-regulated expression of *mexX*, a component of the RND-type multidrug efflux transporter MexXY-OprM (Fig. 1C). Two types of ciprofloxacin-resistant mutants were obtained: one (CIP131) showed increased MIC for fluoroquinolones (ciprofloxacin and levofloxacin) only due to a mutation in GyrA, a subunit of DNA gyrase (Table 3, 5). The other (CIP101 and CIP126) showed resistance not only to fluoroquinolones, but also tetracyclines, chloramphenicol, erythromycin, and acriflavine. These drugs were previously reported to be exported via the RND-type multidrug efflux transporter MexCD-OprJ^4,12^, and the up-regulated expression of *mexCD-oprJ* was observed (Fig. 1B). The two CIP mutant types were used as the parental strain in the next step. Mutants were exposed to eight different drug sequences.

**Table 3:** Drug susceptibility test for mutants from *P. aeruginosa.*

Strain	MIC (μg/ml)									
	CAR	IPM	GEN	AMK	CIP	LVX	TET	CHL	ERY	ACR
PAO1	32	1	4	4–8	0.25	0.5–1	32	64	256	128
GM458	32	1	32	64	1	4	32	32	512	128
GI4401	32	16	32	64	1	4	32–64	32	512	128
GIC44801	32	16	32	64	8	8	32	32	256	128
GC4801	32	1	32	64	16	16	64	32	512	128
GCI48410	32	16	32	64	16	16	64	32	512	256
IPM429	32	16	4	8	0.25	0.5	32	64	256	128
IC4430	16	16	2	2	4	8	64	256	1024	4096
ICG444391	16	16	32	32	4	4	64	128	1024	4096
IG4455	16	16	64	64	1	2	32–64	32–64	256–512	128
IGC448001	16	16	32–64	64	8	8	32–64	32–64	256	128
CIP101	8	0.5	1	1	4	8	64	256	1024	4096
CG4401	4	0.5	16	32	4	4	64	32	1024	4096
CGI44201	4	8	8–16	32	4	8	32–64	64	1024	4096
CIP126	16	1	2	2	4	8	64	128	1024	4096
CI4401	8–16	16	1	1	4	4	64	256	1024	4096
CIG44408	4–8	16	16	32	4	4–8	3–64	128	512–1024	4096
CIP131	32	1	4	8	4	8	32	32	256	64
CgG4479	16	1	64	64	16	32	32	32	256	128
CgGI44405	8–16	16	32	32–64	16–32	16	64	32	256	64–128
CgI4401	32	16	4	8	4	8	32	32	256	64
CgIG44441	16	16	64	64	8	16	64	64	512	128

ACR: acriflavine, AMK: amikacin, CAR: carbenicillin, CHL: chloramphenicol, CIP: ciprofloxacin, ERY: erythromycin, GEN: gentamicin,

IPM: imipenem, LVX: levofloxacin, TET: tetracycline.

### Isolation of mutants from GM458

GI mutants were isolated from GM458 exposed to 4 μg/mL of imipenem. Five mutants showed increased MIC for imipenem only (8- to 32-fold) (Tables 3 and S1). In the representative GI4401, the OprD protein was not detected in the membrane fraction (Fig. 3). In this strain, a one-nucleotide deletion was observed in the *oprD* coding region, leading to a frameshift mutation (Table 4). Ciprofloxacin resistant mutants were isolated from GI4401 exposed to 2 μg/ml of ciprofloxacin. MIC for ciprofloxacin and levofloxacin increased in ten randomly selected mutants (4- to 16- and 8- to 16-fold, respectively) (Tables 3 and S1). All ten mutants satisfied the criteria for MDRP. Except for GIC44805, mutations in the quinolone resistance-determining region (QRDR) of *gyrA* or *gyrB* were identified. (Table 5).

**Table 4:** Mutation in *oprD.*

Strain	Position	Nucleotide change	Protein change
IPM429	1147	G deletion	Frameshift
GI4401	631	G deletion	Frameshift
CI4401	1017	G to A	Non-sense
CgI4401	904	C deletion	Frameshift
GCI48410	1041	A insertion	Frameshift
CGI44201	893–1194	302 base deletion	Truncation and Frameshift
CgGI44405	1115	T deletion	Frameshift

**Table 5:** Mutations in the Quinolone Resistance-determining Region.

Group	Strain	Protein	Amino acid change
C	CIP131	GyrA	Thr83Ile
GC	4803, 4804, 4809, 4810, 4817, 4821	GyrA	Asp82Asn
	4813	GyrA	Thr83Ile
	4801	GyrA	Asp87Asn
	4805, 4815	GyrA	Asp87Gly
GIC	44801, 44802, 44807, 44810, 44814, 44819	GyrA	Asp87Gly
	44809, 44817	GyrA	Asp87Tyr
	44812	GyrB	Ser466Tyr
IGC	448001, 448005, 448011, 448020, 448030	GyrB	Ser466Phe
	448056, 448072, 448080, 448092, 448114		

We isolated ciprofloxacin resistant mutants (GC mutants) from GM458 exposed to 2 μg/ml of ciprofloxacin. Ten of the mutants showed increased MIC for ciprofloxacin and levofloxacin, but not for any other drugs (Tables 3 and S2). This result indicated that these mutants were DNA gyrase mutants, but not MexCD-OprJ up-regulated mutants. All mutants had a mutation in the QRDR of *gyrA* (Table 5). Imipenem resistant mutants (GCI mutants) were isolated from GC4801 exposed to 4 μg/mL of imipenem. All ten GCI mutants exhibited increased resistance to imipenem (16- to 32-fold increase as GC4801) (Table 3). In GCI48410, an insertion mutation was observed in *oprD*, resulting in a frameshift mutation (Table 4), and the OprD protein was not detected in the outer membrane fraction (Fig. 3). All ten GCI mutants satisfied the criteria for MDRP (Table S2).

### Isolation of mutants from IPM429

Ciprofloxacin-resistant mutants (IC mutants) were isolated by exposure of IPM429 to 1 μg/ml ciprofloxacin. Ten IC mutants showed increased MIC not only for fluoroquinolones, but also for chloramphenicol, erythromycin, and acriflavine (Tables 3 and S3). The up-regulation of *mexCD-oprJ* appeared to have occurred. In six out of the ten mutants, MIC for carbenicillin, imipenem, gentamicin, and amikacin were decreased (Table S3). A similar phenomenon was reported for the *nfxB* mutant^13,14^. The mRNA level of *mexC* was increased in IC4430, whereas the MICs of carbenicillin, imipenem, gentamicin, amikacin were unchanged. In IC4404, the MICs for carbenicillin, imipenem, gentamicin, amikacin were decreased (Fig. 1B). Using IC4430, gentamicin resistant mutants (ICG mutants) were isolated by an exposure to 16 μg/ml of gentamicin. All ten ICG mutants showed significantly increased MIC for gentamicin and amikacin, whereas nine showed decreased MIC for imipenem, ciprofloxacin, levofloxacin, tetracycline, chloramphenicol, and erythromycin (Tables 3 and S3). In ICG444391, which maintained MIC for imipenem, ciprofloxacin, levofloxacin, tetracycline, chloramphenicol, and erythromycin, *mexX* was highly expressed in ICG444391 (Fig. 1D). Only ICG444391 satisfied the criteria for MDRP.

Gentamicin resistant mutants (IG mutants) were isolated from IPM429 exposed to 16 μg/ml of gentamicin. Ten mutants showed increased resistance to gentamicin and amikacin (8- to 16- and 4- to 8-fold, respectively) (Tables 3 and S4). These drug resistant patterns indicated the up-regulated expression of *mexXY*; however, three mutants (IG4405, IG4427, and IG4485) were found to easily return to the expression levels of the parental strain with a subculture in broth without antibiotics. We selected IG4455 showing stable resistance for subsequent analyses. In IG4455, the expression of *mexX* was up-regulated (Fig. 1D). Ciprofloxacin resistant mutants (IGC mutants) were isolated from IG4455 exposed to 2 μg/ml of ciprofloxacin. Ten mutants exhibited increased resistance to ciprofloxacin and levofloxacin (Table S4), and the GyrB mutation was identified in the QRDR (Ser466Phe) of all mutants (Table 5).

### Mutants isolated from CIP101 and CIP126

The up-regulated expression of *mexC* was observed in CIP101 (Fig. 1B). Gentamicin resistant mutants (CG mutants) were isolated following an exposure to 16 μg/ml of gentamicin. Ten mutants showed increased MIC for gentamicin and amikacin (8- to 64-fold) (Tables 3 and S5). In CG4401, the expression of *mexX* was up-regulated (Fig. 1D). Imipenem resistant mutants (CGI mutants) were isolated from CG4401 with 2 μg/ml of imipenem, and ten mutants showed increased MIC for imipenem only (8-to 16-fold) (Tables 3 and S5). The OprD protein was not detected in the membrane fraction of CGI44201 (Fig. 3). A large deletion was identified in the *oprD* coding region (Table 4). All ten CGI mutants were defined as non-MDRP because the MIC for imipenem was lower than 16 μg/ml.

We attempted to isolate imipenem resistant mutants (CI mutants) from CIP101 exposed to 2 μg/ml of imipenem, but were unsuccessful for unknown reasons. However, CI mutants were isolated from CIP126, a mutant with the up-regulated expression of *mexC*, exposed to 4 μg/ml of imipenem. All ten CI mutants showed a 16-fold higher MIC for imipenem (Tables 3 and S6). The OprD protein was not detected in the membrane fraction of CI4401 (Fig. 3), and a nonsense mutation was identified in the *oprD* coding region (Table 4). Gentamicin resistant mutants (CIG mutants) from CI4401 isolated with 16 μg/ml of gentamicin showed increased resistance to gentamicin and amikacin (Tables 3 and S6). One of these mutants, CIG44408 showed the up-regulated expression of *mexX* (Fig. 1E). Three out of the ten CIG mutants (CIG44402, CIG44404, and CIG44408) were categorized as MDRPs.

### Mutants isolated from CIP131

CIP131 exhibited increased resistance to ciprofloxacin and levofloxacin, which indicated that it was a DNA gyrase mutant. We identified a point mutation (Thr83Ile) in the QRDR of GyrA (Table 5). Using 16 μg/ml of gentamicin, we isolated gentamicin resistant mutants (CgG mutants). Nine CgG mutants showed higher MIC than CIP131 for gentamicin, amikacin, ciprofloxacin, and levofloxacin (Tables 3 and S7). The up-regulated expression of *mexX* was observed in CgG4479 (Fig. 1D). Imipenem resistant mutants (CgGI mutants) were isolated from CgG4479 exposed to 4 μg/ml of imipenem. All ten CgGI mutants showed 8- to 16-fold higher MIC for imipenem only (Tables 3 and S7). Five out of the ten mutants were recognized as MDRPs. One nucleotide deletion in *oprD*, resulting in a frameshift mutation, was identified in CgGI44405 (Table 4), while the OprD protein was not detected in the outer membrane fraction (Fig. 3).

Imipenem resistant mutants (CgI mutants) from CIP131 were isolated with 4 μg/ml of imipenem. All ten CgI mutants showed increased resistance to imipenem only (Tables 3 and S8), and a one-nucleotide deletion (and a frameshift mutation) was identified in *oprD* in CgI4401 (Table 4). The OprD protein was not detected by an immunoblotting analysis (Fig. 3). Gentamicin resistant mutants (CgIG mutants) were isolated with 16 μg/ml of gentamicin. These mutants showed increased MIC for gentamicin, amikacin, ciprofloxacin, and levofloxacin (Tables 3 and S8). Higher expression of *mexX* was observed in CgIG44441 (Fig. 1D). Eight mutants were categorized as MDRP.

### Appearance rate of MDRP with sequential exposure to drugs

We considered the appearance rate of MDRP according to MIC for ciprofloxacin, gentamicin and imipenem. In the case of GCI, GIC, and IGC mutants, all mutants were categorized as MDRPs. Five out of ten CgGI mutants showed MIC of 8 μg/ml for imipenem, while the remaining five showed 16 μg/ml. Since this difference was only two-fold, it was considered to be negligible. The sequential order of GCI, GIC, IGC, and CgGI increased the appearance rate of MDRP.

MDRPs were not included in CGI mutants. Among the ICG, CIG, and CgIG mutants, some strains were MDRPs. However, many MDRPs exhibited unstable resistance to gentamicin. One and three MDRPs were initially included in ICG and CIG mutants, respectively; however, resistance to gentamicin disappeared after the subculture. In CgIG, although eight isolates initially showed the MDRP phenotype, seven were unstable MDRPs.

## Discussion

We herein attempted to clarify the mechanisms contributing to the emergence of MDRPs. As expected, we were unable to isolate MDRPs following an exposure to only one drug. We also did not isolate any MDRPs following a simultaneous exposure to three drugs, so far. If the mutant appearance frequency for each drug is 10^-8^, triple resistance frequency will be 10^−24^. This probability is not zero, but is almost impossible to achieve in a laboratory or clinical setting. On the other hand, MDRPs were obtained after a sequential exposure to each drug. As reported by many clinicians and pharmacists, the sequential use of different types of drugs promoted the emergence of MDRP with a higher risk.

The order of drug exposure affected the emergence of MDRPs. An exposure to gentamicin before ciprofloxacin resulted in a higher appearance rate of MDRPs. This result indicated that the mechanisms underlying ciprofloxacin resistance are related to the appearance rate of MDRP. After the initial exposure to ciprofloxacin, the MICs of chloramphenicol, erythromycin, and acriflavine, as well as ciprofloxacin and levofloxacin, increased. Such resistance patterns were consistent with the transport substrates for MexCD-OprJ. As revealed by RT-PCR (Fig. 1B), the *mexC* expression significantly increased in CIP126. A similar phenomenon was observed for IC mutants from IPM429. These results indicated increases in the expression of *mexC* in cells exposed to ciprofloxacin before the up-regulated expression of *mexXY*. On the other hand, the expression of *mexXY* was already up-regulated in ciprofloxacin-treated cells exposed to gentamicin, and a mutation in DNA gyrase was detected in all mutants (GC, IGC, and GIC mutants). Therefore, the order of exposure to antimicrobial agents may affect the resistance mechanism, namely, a gyrase mutation or the higher expression of *mexCD-oprJ*, with stable MDRPs emerging with specific orders.

Several mechanisms of aminoglycoside resistance in *P. aeruginosa* have been reported to date, such as inactivation with modifying enzymes, mutations in ribosomes, and the hyperexpression of *mexXY*^15^. Many mutants showed the hyperexpression of *mexXY* in the present study. Furthermore, mutations related to the hyperexpression of *mexXY* have been reported^7,16–26^. Although we have not yet identified nucleotide changes for gentamicin resistance in our mutants, they may provide some important insights into the frequency of MDRPs.

Unstable gentamicin-resistant mutants were also isolated at a certain frequency at every step in the isolation of gentamicin mutants. This may be a type of ‘adaptive resistance’^27^. In our experiments, stable gentamicin mutants were selected for the 2nd or 3rd steps. Very few stable gentamicin mutants were isolated in the 3rd step; therefore, the stability of gentamicin resistance may also be a key factor contributing to the incidence of MDRPs.

The acquisition of exogenous genes may be important as a mechanism for the emergence of MDRPs at clinical sites. The modification enzyme for gentamicin resistance and the metallo β-lactamase for imipenem resistance need to be considered. Since the acquisition of exogenous genes cannot occur in the laboratory, the present results may not directly reflect the mechanisms underlying the emergence of MDRP under clinical settings. However, the results obtained indicated that risk factors for the appearance of MDRP are (1) stable gentamicin resistance and (2) an exposure to gentamicin before ciprofloxacin. If ciprofloxacin is prescribed to treat infections with strains that developed stable gentamicin resistance by incorporating exogenous gentamicin resistance genes, the frequency of MDRP may markedly increase. Revealing the prevalence of exogenous gentamicin resistance genes in clinical settings may provide a whole picture of the emergence of MDRP.

## Conclusion

We showed the appearance rate of MDRP varied depending on the order of exposure; MDRPs more frequently appeared when gentamicin was applied before ciprofloxacin, but were rarely isolated when ciprofloxacin was applied first. And exposure to ciprofloxacin followed by gentamicin increased the expression of MexCD-OprJ, an RND-type multidrug efflux pump, due to the NfxB mutation. In contrast, exposure to gentamicin followed by ciprofloxacin resulted in more mutations in DNA gyrase. These results suggest that the type of quinolone resistance mechanism is related to the frequency of MDRP and that the risk of MDRP incidence is highly dependent on the order of exposure to gentamicin and ciprofloxacin.

## Materials and methods

### Isolation of drug-resistant mutants

Cells of *P. aeruginosa* PAO1 or mutants (10^7^ to 10^9^ CFU) were grown in L medium (1.0% polypeptone, 0.5% yeast extract, and 0.5% NaCl, pH 7.0) and spread onto L agar plates (1.0% polypeptone, 0.5% yeast extract, 0.5% NaCl, and 1.5% agar, pH 7.0) containing 1 × , 2 × , 4 × MIC for one of the following seven antimicrobial agents: carbenicillin, imipenem, amikacin, gentamicin, ciprofloxacin, levofloxacin, and erythromycin. We obtained many colonies that appeared on the plates after an incubation at 37 ℃ for 24–36 h. After single-colony isolation, the drug resistance patterns of the mutants were investigated. Amikacin (Wako, cat. 014-24941), carbenicillin (Wako, cat. 037-23693), ciprofloxacin (Wako, cat. 032-18731), gentamicin (Wako, cat. 079-02973), imipenem (Wako, cat. 098-07283), levofloxacin (Fluka, cat. 28266) were purchased from indicated manufactures.

### Drug susceptibility test

The minimum inhibitory concentrations (MIC) for various drugs were assessed in Muller-Hinton broth (Difco) by the two-fold dilution method according to the CLSI recommendations (CLSI, 2006). Cells of the test medium (10^5^ cells ml^−1^) were incubated at 37 °C for 24 h, and growth was then measured.

### RT-PCR

RNA preparations and reverse transcriptional PCR were performed according to the manufacturer’s protocols. Briefly, total bacterial RNA was isolated from cells grown to an OD650 of 0.7 using the RNeasy Mini Kit (Qiagen). Residual DNA was removed by the treatment with RNase-Free DNase (Promega). One nanogram of DNase-treated RNA was used as the template for one reaction using the Qiagen OneStep RT-PCR kit (Qiagen). Primer pairs to detect mRNA are listed in Table S9. The expression of the *rpsL* gene was used as an internal control. PCR cycles were 27 for *mexA*, 33 for *mexC*, 32 for *mexX*, and 24 for *rpsL*. Products were separated using 3% agarose gel (Nippongene) electrophoresis and visualized with ethidium bromide.

### Western blotting for OprD

 Preparation of OprD was performed by previously described method with a slight modification^28^. *P.* *aeruginosa* cells were grown to the mid-log phase (OD650 = 0.7), harvested, and suspended in 50 mM Tris–HCl (pH7.4) + 5 mM MgSO_4_. Cells were broken with the sonicator Vibra cell VC505. Unbroken cells were removed, the membrane fraction was prepared with ultracentrifugation (100,000 × *g*). The pellet was washed twice, and dissolved with same buffer. SDS–polyacrylamide gel electrophoresis was performed as previously described^29^, 40 μg per sample. Proteins were transferred to a nitrocellulose membrane filter (ADVANTEC Toyo). An rabbit anti-OprD antibody was kindly provided by Meiji Seika Pharma Co.^30^. A goat anti-rabbit IgG antibody (Bioss Inc, cat. bs-0295G-HRP) was used as the 2nd antibody, and OprD was detected using the ECL system (Amersham Pharmacia Biotech).

## Supplementary Information

Supplementary Figures.

Supplementary Tables.

## Data Availability

The datasets used and/or analyzed during the current study available from the corresponding author on reasonable request.
