# Supplementary material for: Effects of the order of exposure to antimicrobials on the incidence of multidrug-resistant Pseudomonas aeruginosa
Source: Sci Rep. 2023 May 31;13:8826. doi: 10.1038/s41598-023-35256-8 (PMC10232440; doi:10.1038/s41598-023-35256-8)
Supplement: Supplementary file 1 — Supplementary Figures. [file 41598_2023_35256_MOESM1_ESM.pdf]

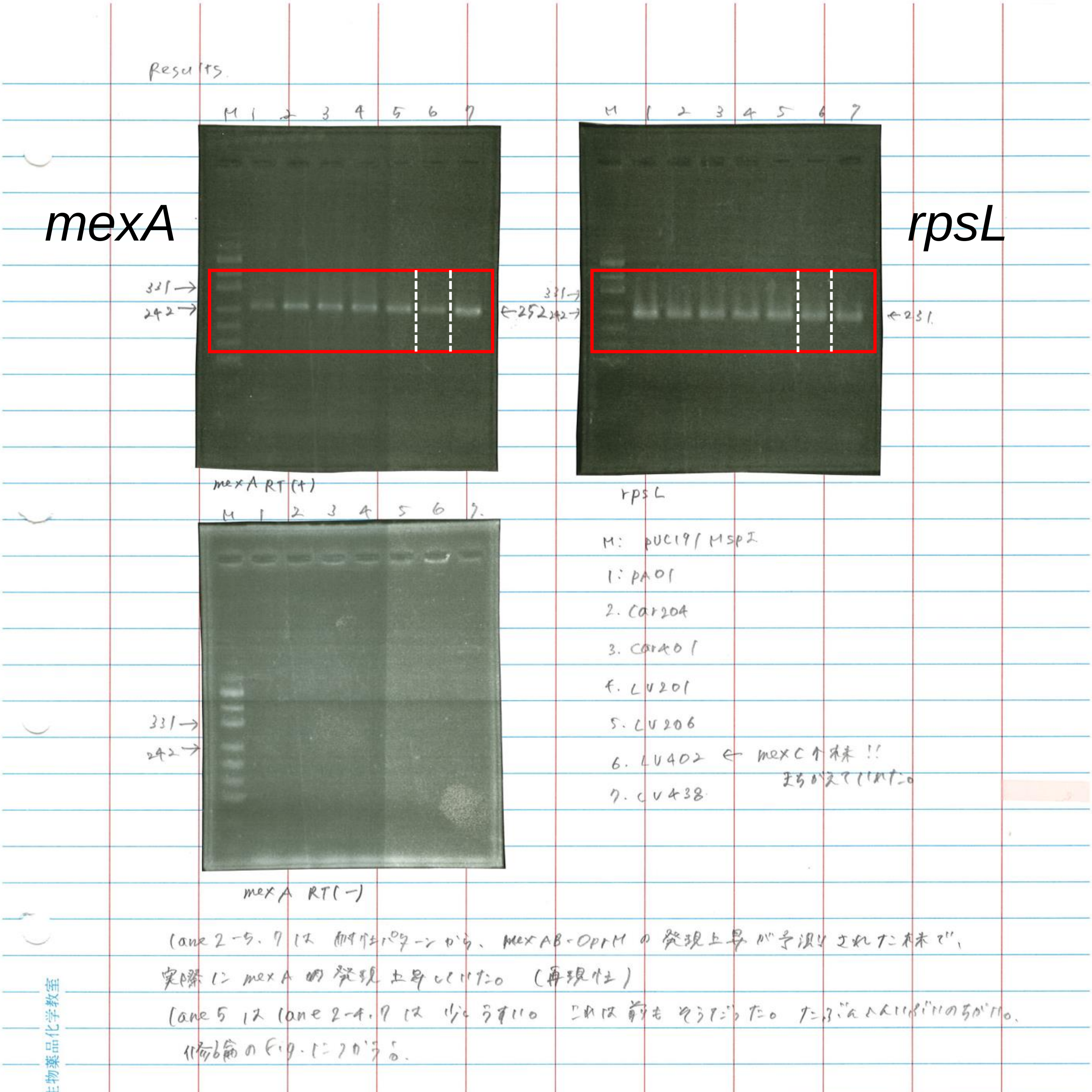

Fig. 1A(Supplemental)
Gels stained with ethidium bromide and UV irradiated were printed on thermal paper. The images were scanned and converted to image data. The white dashed line indicated a cut section of a photograph of electrophoresis. The area indicated by the red frame corresponded to Fig. 1(A).

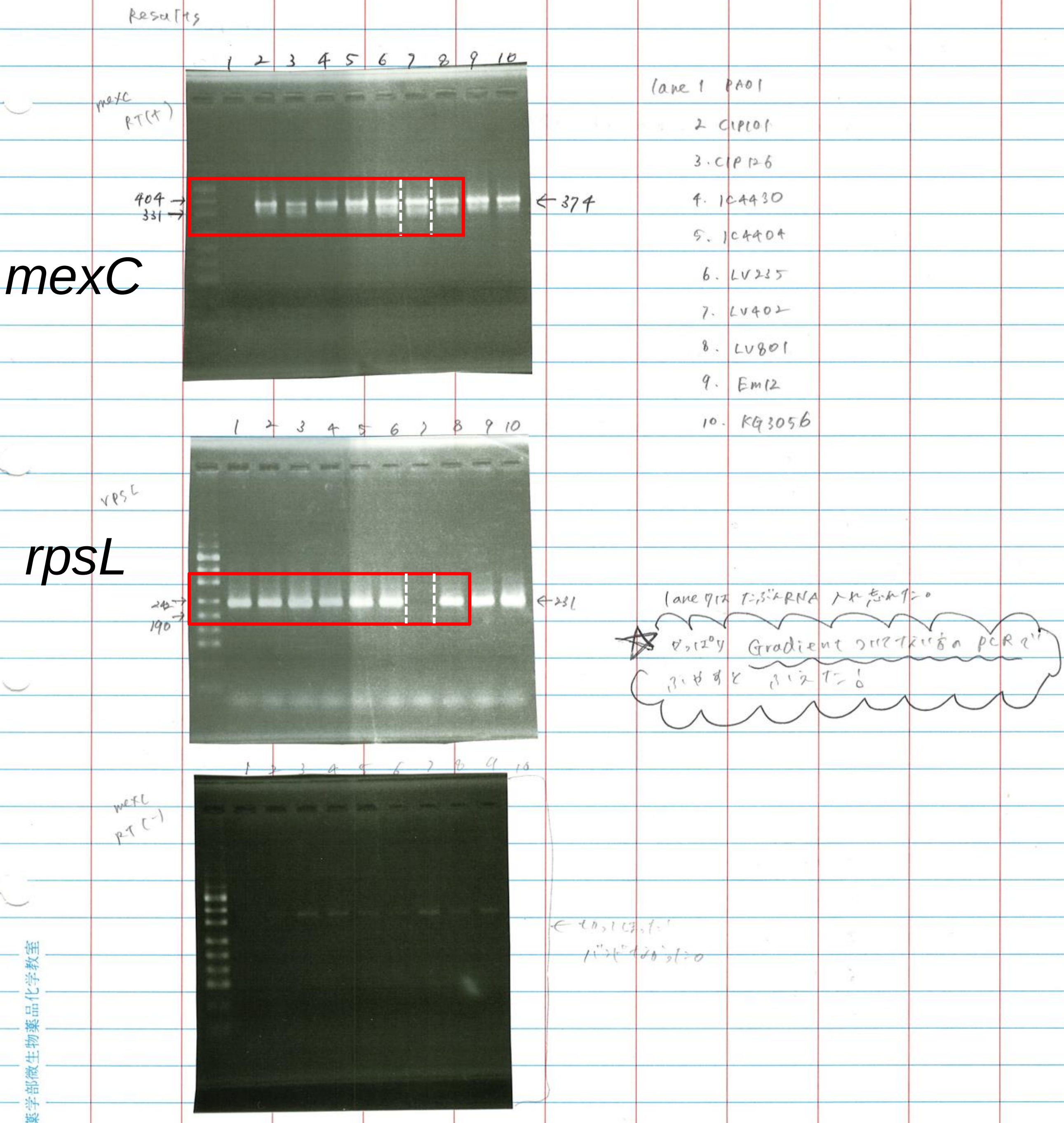

Fig. 1B(Supplemental)
Gels stained with ethidium bromide and UV irradiated were printed on thermal paper. The images were scanned and converted to image data. The white dashed line indicated a cut section of a photograph of electrophoresis. The area indicated by the red frame corresponded to Fig. 1(B).

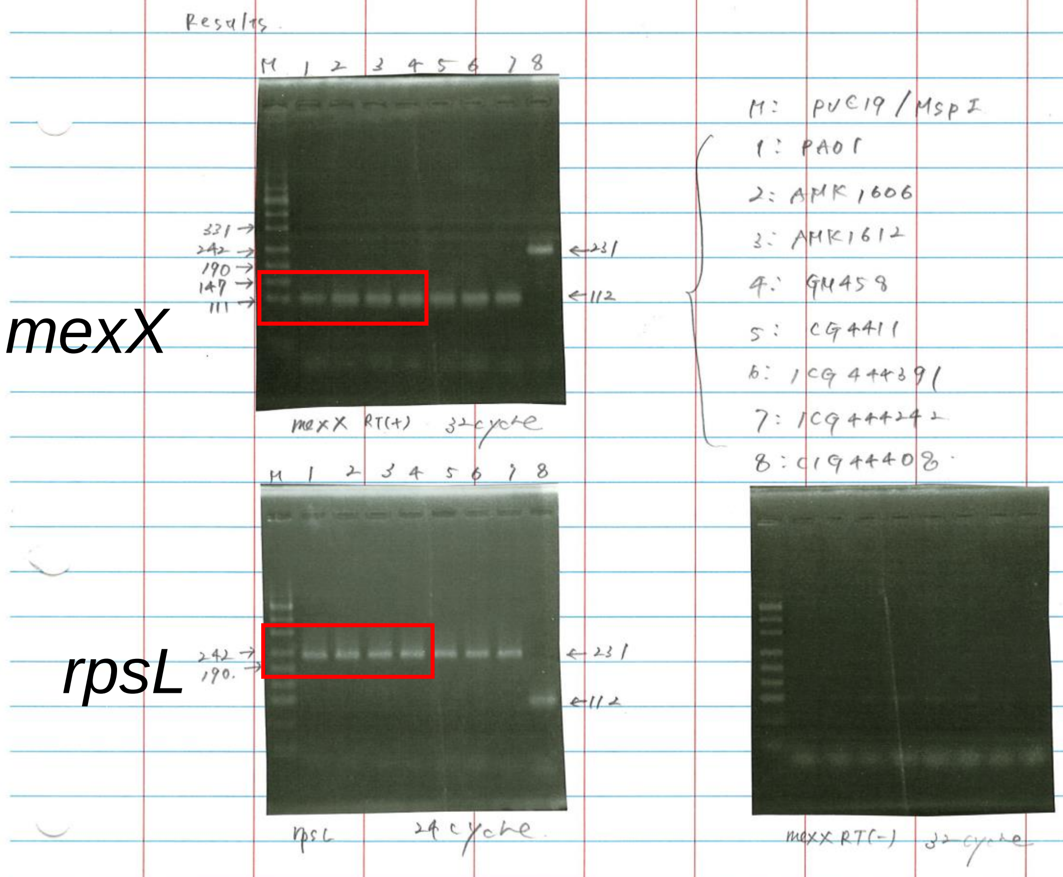

lane 8 のみ mexX RT(+) のサンプルと rpsL のサンプルと入れ替えて流してしまった。
lane 4-8 は再現性とれてる。
lane 2, 3 は リボソームの変異が mexX を発現上昇か!? と思っていた株。
やはり mexX を ↑ らしい。
しかし、この2株では キノロンのMIC が ↑ していない。

Fig. 1C(Supplemental)
Gels stained with ethidium bromide and UV irradiated were printed on thermal paper. The images were scanned and converted to image data. The area indicated by the red frame corresponded to Fig. 1(C).

岡山大学薬学部微生物薬品化学教室

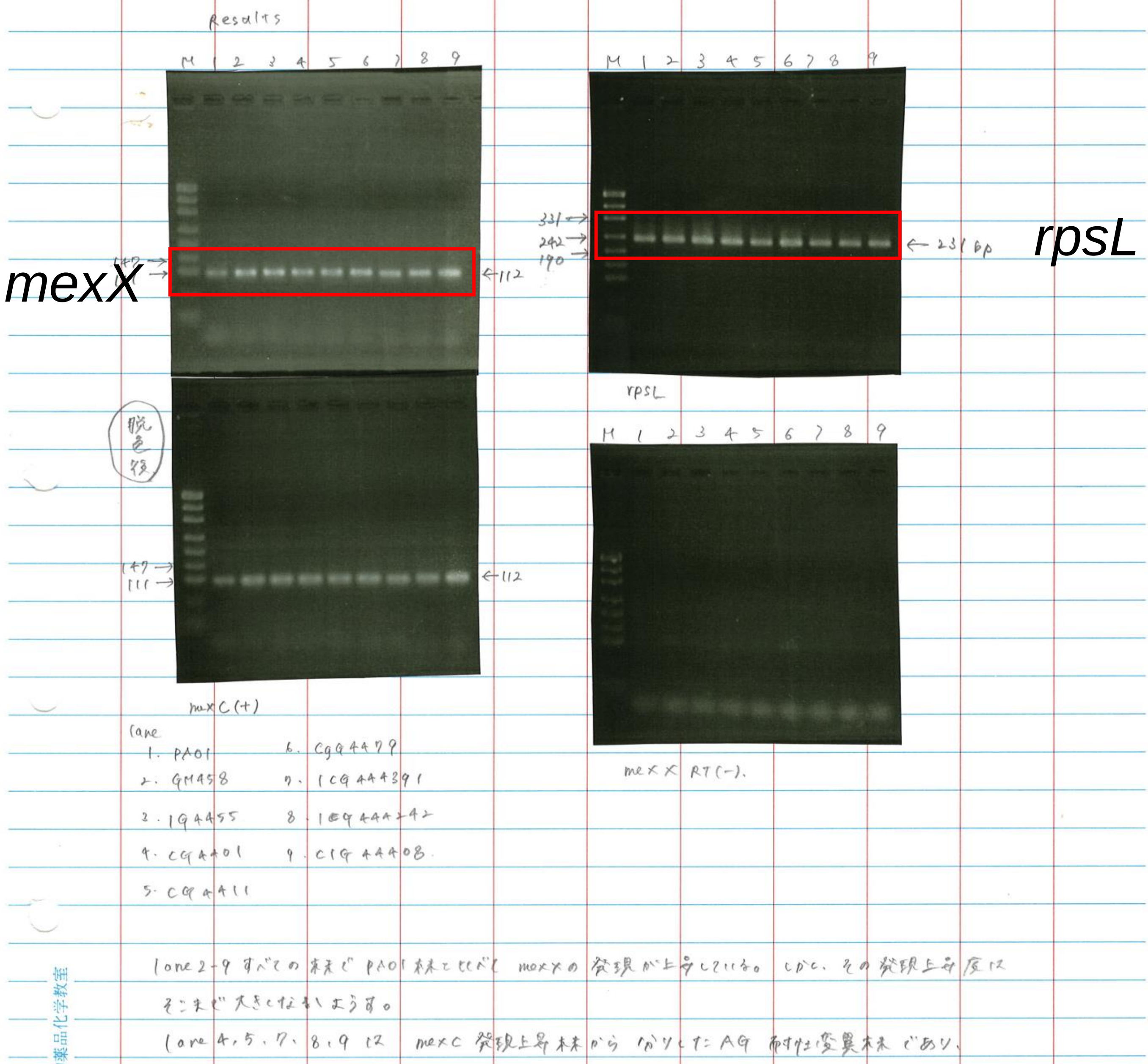

Fig. 1D(Supplemental)
Gels stained with ethidium bromide and UV irradiated were printed on thermal paper. The images were scanned and converted to image data. The area indicated by the red frame corresponded to Fig. 1(D).

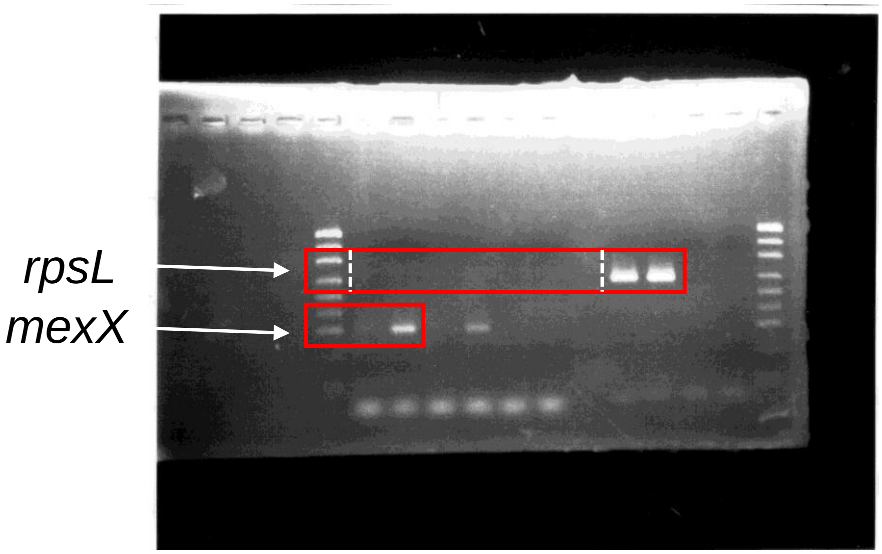

Fig. 1E(Supplemental)
Gel image stained with ethidium bromide and UV irradiated was saved as image file. The white dashed line indicated a cut section of a image of electrophoresis. The area indicated by the red frame corresponded to Fig. 1(E).

Fig. 3 Supplemental

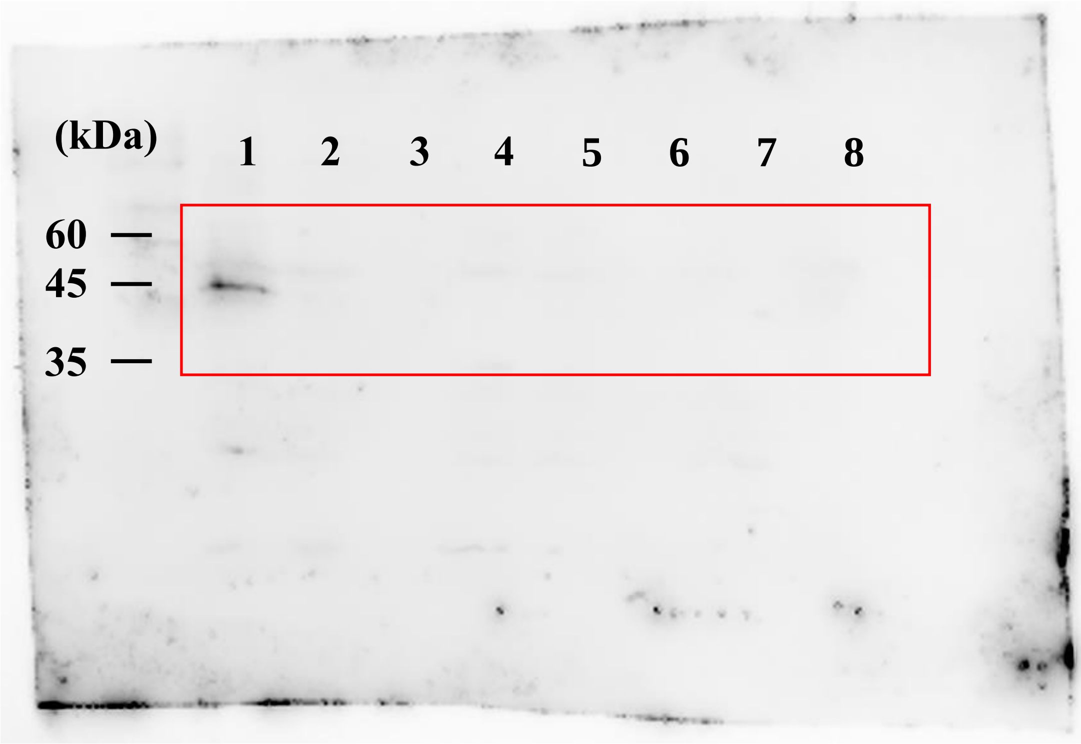

After chemiluminescence treatment, blots were visualized with a gel imaging system. The area indicated by the red frame corresponded to Fig. 3.

1: PAO1, 2: IPM429, 3: GI4401, 4: CI4401, 5: CgI4401,
6: GCI48410, 7: CGI44201, 8: CgGI44405
