# Supplementary material for: Effects of the order of exposure to antimicrobials on the incidence of multidrug-resistant Pseudomonas aeruginosa
Source: Sci Rep. 2023 May 31;13:8826. doi: 10.1038/s41598-023-35256-8 (PMC10232440; doi:10.1038/s41598-023-35256-8)
Supplement: Supplementary file 2 — Supplementary Tables. [file 41598_2023_35256_MOESM2_ESM.docx]

Table S1 Drug susceptibility test for mutants from *P. aeruginosa* (GIC mutants)

strain MIC (μg/ml)

CAR IPM GEN AMK CIP LVX TET CHL ERY ACR

PAO1 32 1 4 4-8 0.25 0.5-1 32 64 256 128

GM458 32 1 32 64 1 4 32 32 512 128

GI4401 32 16 32 64 1 4 32-64 32 512 128

GI4402 32 8 32 32 0.5-1 2 16-32 16-32 256-512 64

GI4403 32 16 32 64 1-2 4 32-64 32 256 128

GI4414 32 16-32 32 64 1-2 4 32-64 32 256 128

GI4429 32 16-32 32 64 1-2 4 32-64 32 256 128

GIC44801 32 16 32 64 8 8 32 32 256 128

GIC44802 32 16 32 64 8 16 32 16 256 128

GIC44805 32 16 32 64 8 8 32 32 256 128

GIC44807 32 16 32 64 8 8 32 32 256 128

GIC44809 32 16 32 64 8 8 32 32 256 128

GIC44810 32 16 32 64 8 8 32 32 256 128

GIC44812 32 16 32 64 8 8 32 32 256 128

GIC44814 32 16 32 64 8 8 32 32 256 128

GIC44817 32 16 32 64 8 8 32 32 256 128

GIC44819 32 16 32 64 8 8 32 32 256 128

ACR: acriflavine, AMK: amikacin, CAR: carbenicillin, CHL: chloramphenicol, CIP: ciprofloxacin, ERY: erythromycin, GEN: gentamicin,

IPM: imipenem, LVX: levofloxacin, TET: tetracycline

Table S2 Drug susceptibility test for mutants from *P. aeruginosa* (GCI mutants)

strain MIC (μg/ml)

CAR IPM GEN AMK CIP LVX TET CHL ERY ACR

PAO1 32 1 4 4-8 0.25 0.5-1 32 64 256 128

GM458 32 1 32 64 1 4 32 32 512 128

GC4801 32 1 32 64 16 16 64 32 512 128

GC4803 16 1 32 64 16 16 64 16 256 128

GC4804 32 1 32 64 16 16 64 16 256 128

GC4805 32 1 32 64 8 8 64 32 512 128

GC4809 32 2 32 64 16 16 64 32 512 128

GC4810 32 1 32 64 16 16 64 16 512 128

GC4813 32 1 32 64 16 32 64 32 512 64

GC4815 32 1 32 64 8 8 64 32 512 128

GC4817 32 1 32 64 8 16 64 16 256 128

GC4821 32 1 32 64 16 16 64 32 512 128

GCI48401 32 32 32 64 16 16 64 32 512 256

GCI48408 16 16 32 64 16 16 32 32 512 256

GCI48410 32 16 32 64 16 16 64 32 512 256

GCI48416 32 16 32 64 16 16 32 32 512 256

GCI48421 32 16 32 64 16 16 32 32 512 256

GCI48425 16 16 32 64 8 16 32 32 512 256

GCI48426 32 16 32 64 16 16 32 32 512 256

GCI48434 32 32 32 64 16 16 32 32 512 256

GCI48447 32 16 32 64 16 16 32 32 512 256

GCI48449 32 16 32 64 8 16 32 32 512 256

ACR: acriflavine, AMK: amikacin, CAR: carbenicillin, CHL: chloramphenicol, CIP: ciprofloxacin, ERY: erythromycin, GEN: gentamicin,

IPM: imipenem, LVX: levofloxacin, TET: tetracycline

Table S3 Drug susceptibility test for mutants from *P. aeruginosa* IPM429 (ICG mutants)

strain MIC (μg/ml)

CAR IPM GEN AMK CIP LVX TET CHL ERY ACR

PAO1 32 1 4 4-8 0.25 0.5-1 32 64 256 128

IPM429 32 16 4 8 0.25 0.5 32 64 256 128

IC4430 16 16 2 2 4 8 64 256 1024 4096

IC4401 8 8 1 1 4 8 64 256 1024 4096

IC4404 8 2 1 1 4 8 64 256 1024 4096

IC4405 4 8 1 1 4 8 64 256 1024 4096

IC4415 4 2 1 1 4 8 64 256 1024 4096

IC4417 16 16 2 2 2-4 8 64 256 1024 4096

IC4420 4 2-4 1 1 4 8 64 256 1024 4096

IC4428 8 16 2 2 2 4 64 128-256 1024 4096

IC4432 16 16 2 2 2 4 64 128 1024 4096

IC4435 4 4 2 1 4 8 64 128-256 1024 4096

ICG444041 8 8 16 64 2 2 32 32 512 4096

ICG444081 8 8 32 32 2 1-4 32 32 512 4096

ICG444201 16 16 32 32 2 2 64 64-128 512-1024 4096

ICG444231 8 8 32 64 2 2 32-64 32 512 4096

ICG444261 8 8 16 64 2 2 32 16-32 512 4096

ICG444311 8 8 16 64 2 2 32 16-32 512 4096

ICG444391 16 16 32 32 4 4 64 128 1024 4096

ICG444401 8 8 16 32 2 2 32 16-32 512 4096

ICG444451 4 8 8-32 32 2 2 32 32 512 4096

ICG444521 8 8 16 32 2 4 32-64 128 1024 4096

ACR: acriflavine, AMK: amikacin, CAR: carbenicillin, CHL: chloramphenicol, CIP: ciprofloxacin, ERY: erythromycin, GEN: gentamicin,

IPM: imipenem, LVX: levofloxacin, TET: tetracycline

Table S4 Drug susceptibility test for mutants from *P. aeruginosa* IPM429 (IGC mutants)

strain MIC (μg/ml)

CAR IPM GEN AMK CIP LVX TET CHL ERY ACR

PAO1 32 1 4 4-8 0.25 0.5-1 32 64 256 128

IPM429 32 16 4 8 0.25 0.5 32 64 256 128

IG4405 32 16 32 64 1 2 32 32 256 128

IG4411 32 16 64 64 1 4 32 32 256 128

IG4420 32 16 64 64 1 4 32 32 256 128

IG4426 32 16 32 64 1 2 32 32 256 128

IG4427 32 16 32 64 0.5 2 32 32 256 128

IG4455 16 16 64 64 1 2 32-64 32-64 256-512 128

IG4461 32 16 64 64 2 4 64 32 256 128

IG4463 32 16 32 64 2 4 64 64 256 128

IG4476 16 16 64 64 2 2 64 32 256 128

IG4485 32 16 32 8-32 0.25 1 64 64 256 128

IGC448001 16 16 32-64 64 8 8 32-64 32-64 256 128

IGC448005 16 16 32-64 64 4-8 8 32-64 32-64 256 128

IGC448011 16 16 32 64 4-8 8 32-64 32-64 256 128

IGC448020 16 16 32 64 4-8 8 32-64 32-64 256 128

IGC448030 16 16 32 64 4-8 8 32-64 32-64 256 128

IGC448056 16 16 32 64 4-8 8 32-64 32-64 256 128

IGC448072 16 16 32-64 64 4-8 8 32-64 32-64 256 128

IGC448080 16 16 32 64 4-8 8 32-64 32-64 256 128

IGC448092 8-16 16 32-64 64 4-8 8 32-64 32-64 256 128

IGC448114 16 16 32-64 64 4-8 8 32-64 32-64 256 128

ACR: acriflavine, AMK: amikacin, CAR: carbenicillin, CHL: chloramphenicol, CIP: ciprofloxacin, ERY: erythromycin, GEN: gentamicin,

IPM: imipenem, LVX: levofloxacin, TET: tetracycline

Table S5 Drug susceptibility test for mutants from *P. aeruginosa* CIP101 (CGI mutants)

strain MIC (μg/ml)

CAR IPM GEN AMK CIP LVX TET CHL ERY ACR

PAO1 32 1 4 4-8 0.25 0.5-1 32 64 256 128

CIP101 8 0.5 1 1 4 8 64 256 1024 4096

CG4401 4 0.5 16 32-64 2-4 4 64 32 1024 4096

CG4402 8 0.5 8 16 4 8 64 256 1024 4096

CG4403 4 1 16 32 4 4 64 64 1024 4096

CG4405 4 1 16 32 4 4 64 32 1024 4096

CG4406 4 1 16 32 4 4 32 32 1024 4096

CG4407 4 1 16 32 4 4 64 32 1024 4096

CG4410 4 1 16 32 4 4 32 32 1024 4096

CG4411 2 0.5 16 32 4 4 32 16 1024 4096

CG4413 2-4 0.5 16 16-32 4 4-8 32 64 512-2048 4096

CG4415 2-4 0.5-1 16 32 4-8 4-8 32-64 32-64 1024-2048 4096

CGI44201 4 8 8-16 32 4 8 32-64 64 1024 4096

CGI44202 4 8 16 32 4 4-8 32-64 64 1024 4096

CGI44204 4 8 8-16 32 4 8 32-64 64 1024 4096

CGI44208 4 8 8-16 32 4 8 32-64 64 1024 4096

CGI44210 4 8 16 32 4 8 32-64 64 1024 4096

CGI44213 4 8 8-16 32 4 8 32-64 64 1024 4096

CGI44214 4 8 16 32 4 8 32-64 64 1024 4096

CGI44225 4 8 16 32 4 8 32-64 64 1024 4096

CGI44228 4 8 8-16 32 4 4-8 32-64 64 1024 4096

CGI44230 4 8 8-16 32 4 4-8 32-64 32-64 1024 4096

ACR: acriflavine, AMK: amikacin, CAR: carbenicillin, CHL: chloramphenicol, CIP: ciprofloxacin, ERY: erythromycin, GEN: gentamicin,

IPM: imipenem, LVX: levofloxacin, TET: tetracycline

Table S6 Drug susceptibility test for mutants from *P. aeruginosa* CIP126 (CIG mutants)

strain MIC (μg/ml)

CAR IPM GEN AMK CIP LVX TET CHL ERY ACR

PAO1 32 1 4 4-8 0.25 0.5-1 32 64 256 128

CIP126 16 1 2 2 4 8 64 128 1024 4096

CI4401 8-16 16 1 1 4 4 64 256 1024 4096

CI4402 8-16 16 1 1 4 4 64 128-256 1024-2048 4096

CI4405 16 16 1-2 1 4 4-8 64 128-256 1024 4096

CI4408 16 16 1 2 4 8 64 256 1024 4096

CI4410 8-16 16 1 1 4 4-8 64 128-256 1024 4096

CI4412 16 16 1 1 4 8 64 256 1024 4096

CI4413 16 16 1 1 4 4 64 256 1024 4096

CI4414 16 16 1 1 4 8 64 256 1024 4096

CI4416 16 16 1 1 4 4 64 256 1024 4096

CI4417 8 16 1-2 1 4 4-8 64 128-256 1024-2048 4096

CIG44401 4 8 16-64 32-64 2 4 32 32 1024 4096

CIG44402 16 16 8 32 4 4-8 64 256 1024 4096

CIG44404 8-16 16 16 32 4 4 64 128 1024 4096

CIG44405 4 8 16 32 2 4 32 32-64 512 4096

CIG44408 4-8 16 16 32 4 4-8 32-64 128 512-1024 4096

CIG44410 4 8 16 32 4 4 32 32 512 4096

CIG44413 4 8 16 32 4 2-4 32 32 1024 4096

CIG44415 4 8 16 32 4 4 32 32 1024 4096

CIG44423 2 4-8 16 32-64 2-4 4 32 16 256-1024 4096

CIG44424 4 8 16 32 4 4 32-64 32 512 4096

ACR: acriflavine, AMK: amikacin, CAR: carbenicillin, CHL: chloramphenicol, CIP: ciprofloxacin, ERY: erythromycin, GEN: gentamicin,

IPM: imipenem, LVX: levofloxacin, TET: tetracycline

Table S7 Drug susceptibility test for mutants from *P. aeruginosa* CIP131 (CgGI mutants)

strain MIC (μg/ml)

CAR IPM GEN AMK CIP LVX TET CHL ERY ACR

PAO1 32 1 4 4-8 0.25 0.5-1 32 64 256 128

CIP131 32 1 4 8 4 8 32 32 256 64

CgG4401 32 1 32 64 16 32 64 32 256 64

CgG4420 32 1 64 64 8 16 64 64 512 128

CgG4428 32 1 64 64 8 16 64 64 256 128

CgG4434 32 1 64 64 8 16 64 64 512 128

CgG4451 32 1 64 64 16 32 64 64 256 128

CgG4469 32 1 64 64 16 32 64 64 256 128

CgG4477 32 1 32 64 8 32 64 64 256 128

CgG4479 32 1 64 64 16 32 32 32 256 128

CgG44112 32 1 64 64 16 32 32 32 256 64

CgGI44401 16 16 32 32 16 32 64 64 256 128

CgGI44405 8-16 16 32 32-64 16-32 16 64 32 256 64-128

CgGI44410 16 8 32 64 16 32 32 64 256 128

CgGI44418 32 16 32 32 16 32 32 32 256 128

CgGI44420 4-16 8 32 32 16 32 32 32 256 128

CgGI44421 16 16 32 64 16 32 32 64 256 128

CgGI44429 16 8 32 32 16 32 32 32 256 128

CgGI44431 4-16 8 32 32-64 16-32 16-32 32 32 256 128

CgGI44443 4-16 8 32 64 16-32 16-32 32 32-64 256 128

CgGI44445 8-16 16 32 64 16-32 32 32 32 256 128

ACR: acriflavine, AMK: amikacin, CAR: carbenicillin, CHL: chloramphenicol, CIP: ciprofloxacin, ERY: erythromycin, GEN: gentamicin,

IPM: imipenem, LVX: levofloxacin, TET: tetracycline

Table S8 Drug susceptibility test for mutants from *P. aeruginosa* CIP131 (CgIG mutants)

strain MIC (μg/ml)

CAR IPM GEN AMK CIP LVX TET CHL ERY ACR

PAO1 32 1 4 4-8 0.25 0.5-1 32 64 256 128

CIP131 32 1 4 8 4 8 32 32 256 64

CgI4401 32 16 4 8 4 8 32 32 256 64

CgI4402 32 16 4 8 4 8 32 64 512 64

CgI4403 32 16 4 8 4 8 32 64 256 64

CgI4408 32 16 4 8 4 8 32 64 512 64

CgI4410 32 16 4 8 4 8 32 64 256 64

CgI4419 32 16 4 8 4 8 32 64 256 64

CgI4423 32 16 4 8 4 8 32 64 512 64

CgI4425 32 16 8 8 4 8 32 32 256 64

CgI4427 32 16 4 8 4 8 32 64 512 64

CgI4430 32 16 8 8 4 8 32 64 512 64

CgIG44401 32 16 32 32 16 16 32 32 256 64

CgIG44408 32 16 32 64 16 16 32 32 256 64

CgIG44430 32 16 16-32 32-64 8 8-16 32 32-64 256 64

CgIG44441 16 16 64 64 8 16 64 64 512 128

CgIG44445 32 16 32 64 8 8 32 64 256 64

CgIG44451 32 16 32 32-64 16 16 32 64 256 64

CgIG44458 32 16 32 32 8 8 32 64 256 64

CgIG44497 16 8 32 32 8 16 64 32-64 256-512 64-128

CgIG44499 32 16 32 64 16 16 64 64 256 128

CgIG444118 32 8 32 32 8 16 32 32-64 256 64

ACR: acriflavine, AMK: amikacin, CAR: carbenicillin, CHL: chloramphenicol, CIP: ciprofloxacin, ERY: erythromycin, GEN: gentamicin,

IPM: imipenem, LVX: levofloxacin, TET: tetracycline

Table S9 Primers used in the present study

Primer Sequence (5’ to 3’)

mexA-F ACCTACGAGGCCGACTACCAGA

mexA-R GTTGGTCACCAGGGCGCCTT

mexC-F AGCCAGCAGGACTTCGATACC

mexC-R ACGTCGGCGAACTGCAAC

mexX-F TTCCTGCTGGGCTGCGAAGA

mexX-R AGCTCGCTGGTGATGCCGATAG

rpsL-F GCAACTATCAACCAGCTGGTG

rpsL-R GCTGTGCTCTTGCAGGTTGTG
